# Temporary Mechanical Circulatory Support in Acute Myocardial Infarction-Related Cardiogenic Shock: Lessons from Contemporary Randomized Trials

**DOI:** 10.3390/jcm15197664

**Published:** 2026-10-03

**Authors:** Osama Odeh, Mariam Al-Bayati, Ali Abdulrazzaq, Ala W. Abdallah, Kanval Shah

**Affiliations:** 1 Department of Internal Medicine, Houston Methodist The Woodlands Hospital, Conroe, TX 77385, USA; 2 Ottawa Hospital Research Institute, Ottawa, ON K1H 8L6, Canada; 3 Department of Internal Medicine, Brookdale Hospital Medical Center, Brooklyn, NY 11212, USA; 4 Division of Cardiology, LSU Health Sciences Center, Louisiana State University Health Sciences Center, Shreveport, LA 70802, USA; 5 Houston Methodist DeBakey Heart & Vascular Center, Houston Methodist The Woodlands Hospital, Conroe, TX 77385, USA

**Keywords:** cardiogenic shock, acute myocardial infarction, temporary mechanical circulatory support, Impella, VA-ECMO

## Abstract

**Background:** Acute myocardial infarction-related cardiogenic shock (AMI-CS) remains associated with high mortality despite advances in revascularization, critical care, and temporary mechanical circulatory support (MCS). Recent randomized trials have challenged the concept of routine device-based therapy and highlighted the importance of matching MCS strategy to shock phenotype, timing, and patient risk profile. **Methods:** We reviewed contemporary randomized clinical trials and prespecified or post hoc analyses evaluating temporary MCS strategies in adult patients with AMI-CS. PubMed was searched for English-language studies published from January 2021 through June 2026, focusing on intra-aortic balloon pump (IABP), microaxial flow pumps, and venoarterial extracorporeal membrane oxygenation (VA-ECMO/ECLS). Outcomes of interest included mortality, hemodynamic recovery, end-organ outcomes, and device-related complications. **Results:** Recent randomized evidence demonstrates that no temporary MCS device provides universal benefit across the heterogeneous AMI-CS population. Routine IABP use has not been associated with improved survival and is now reserved for selected patients requiring modest circulatory support, bridge-to-decision strategies, or specific mechanical complications. The DanGer Shock trial provided the strongest contemporary randomized evidence supporting temporary MCS, demonstrating reduced 180-day mortality with routine Impella CP support in carefully selected patients with STEMI-related cardiogenic shock. However, this benefit was accompanied by increased major bleeding, limb ischemia, acute kidney injury, and renal replacement therapy. Prespecified secondary and exploratory post hoc analyses have generated hypotheses that treatment response may vary according to age, baseline hemodynamics, timing of support, and shock phenotype; however, these findings require prospective validation and should not be interpreted as established treatment-selection criteria. In contrast, ECLS-SHOCK, ECMO-CS, EURO SHOCK, and randomized meta-analytic data did not demonstrate a survival benefit for routine early VA-ECMO in broad AMI-CS populations and consistently identified higher rates of bleeding and vascular complications. Strategies for routine early left ventricular unloading during VA-ECMO have improved physiologic parameters but have not demonstrated a mortality benefit. **Conclusions:** Contemporary randomized trials have shifted the role of temporary MCS in AMI-CS from routine device deployment toward individualized, phenotype-guided therapy. Impella CP, as evaluated in the DanGer Shock trial, currently provides the strongest randomized survival signal but should be applied selectively in patients with predominantly left ventricular failure treated within experienced shock systems. VA-ECMO remains an important rescue option in carefully selected patients with refractory circulatory collapse, severe hypoxemia, cardiac arrest, or biventricular failure, although routine early use is not supported. Future research should focus on earlier shock phenotyping, physiology-guided device selection, complication reduction, and strategies that integrate mechanical support with recovery-focused care.

## 1. Introduction

Cardiogenic shock is a syndrome of inadequate tissue perfusion caused by cardiac dysfunction and is associated with high early mortality despite contemporary advances in cardiovascular critical care [1]. In acute myocardial infarction-related cardiogenic shock (AMI-CS), abrupt ischemic loss of myocardial function can produce reduced cardiac output, elevated filling pressures, systemic hypoperfusion, lactate accumulation, and multiorgan injury [1].

The Society for Cardiovascular Angiography and Interventions shock classification provides a practical framework for describing shock severity from patients at risk to those in extremis, but shock stage is only one component of risk assessment [2,3,4]. Etiology, hemodynamic phenotype, cardiac arrest status, neurologic injury, age, frailty, and end-organ dysfunction also influence prognosis and treatment selection [1,3].

Early revascularization remains the foundation of therapy in AMI-CS [5]. In patients with multivessel coronary artery disease, current practice generally favors culprit-vessel revascularization during the unstable phase, with staged treatment of nonculprit lesions after hemodynamic stabilization [5,6].

Although the OPTION-STEMI trial did not enroll patients with established cardiogenic shock, it provides only indirect background evidence supporting deferred complete revascularization after successful primary PCI in patients with STEMI and multivessel disease and should not be interpreted as evidence for revascularization strategy in AMI-CS [7]. In contrast, a prespecified exploratory DanGer Shock substudy suggested that, among patients with multivessel coronary artery disease, immediate multivessel PCI was associated with lower observed 180-day mortality than culprit-only PCI; however, because this was a nonrandomized, as-treated analysis, these findings should be interpreted cautiously, considered hypothesis-generating, and viewed in the context of the randomized CULPRIT-SHOCK evidence rather than as evidence establishing a preferred revascularization strategy [8,9].

Residual coronary disease nevertheless remains prognostically relevant, and higher residual SYNTAX scores after PCI have been associated with worse outcomes in patients with myocardial infarction complicated by cardiogenic shock [10]. Contemporary evidence also reinforces the importance of minimizing delays to reperfusion, as patients undergoing primary PCI within 60 min have lower rates of cardiogenic shock and improved short-term outcomes than those treated later, emphasizing the importance of rapid reperfusion before advanced shock physiology becomes established [11].

Temporary mechanical circulatory support (MCS) is intended to interrupt the downward spiral of hypoperfusion, ischemia, congestion, inflammation, and multiorgan dysfunction [1,12,13]. The major temporary MCS platforms used in AMI-CS include intra-aortic balloon pump (IABP) counterpulsation, microaxial flow pumps, and venoarterial extracorporeal membrane oxygenation [12]. These devices are not physiologically interchangeable: IABP provides modest counterpulsation and afterload reduction, microaxial flow pumps directly unload the left ventricle and augment forward systemic flow, and VA-ECMO provides systemic circulatory support and gas exchange but may increase LV afterload unless unloading is added [12,14,15,16].

The central question in contemporary AMI-CS care is therefore not simply whether a device should be used. The more clinically relevant question is which patient, with which shock phenotype, at which point in the disease course, should receive which support strategy.

Although several recent reviews have summarized temporary mechanical circulatory support in cardiogenic shock, many predated the publication of DanGer Shock and its subsequent secondary analyses or did not focus primarily on contemporary randomized evidence. The present narrative review synthesizes contemporary randomized trials together with recent prespecified and post hoc analyses and emphasizes phenotype-guided interpretation, structured comparison of trial characteristics, and clinically relevant complication profiles to support individualized decision-making in AMI-related cardiogenic shock.

## 2. Methods

### Literature Search

This narrative review was informed by a targeted search of PubMed to identify English-language publications from 1 January 2021, through 29 June 2026. Search terms included combinations of “acute myocardial infarction,” “cardiogenic shock,” “mechanical circulatory support,” “intra-aortic balloon pump,” “IABP,” “Impella,” “microaxial flow pump,” “venoarterial extracorporeal membrane oxygenation,” “VA-ECMO,” “extracorporeal life support,” “ECLS,” “randomized trial,” “secondary analysis,” “post hoc analysis,” and “long-term follow-up.” Primary randomized controlled trials formed the foundation of this review. Prespecified secondary analyses and post hoc analyses were included to provide additional clinical context, with post hoc findings interpreted as exploratory and hypothesis-generating. Landmark studies and contemporary guideline documents published before the search period were cited selectively to provide background and contextual interpretation.

As this is a narrative review, the literature was selected to provide a clinically relevant synthesis of contemporary randomized evidence rather than a systematic or exhaustive evidence review. Accordingly, study selection was based on clinical relevance and the objective of providing an up-to-date overview of the current randomized evidence base.

## 3. Device-Specific Physiologic Considerations

### 3.1. Intra-Aortic Balloon Pump

IABP is the oldest and most widely familiar temporary MCS platform [12,13,17]. It is positioned in the descending thoracic aorta and uses counterpulsation: balloon inflation during diastole augments diastolic aortic pressure, while deflation before systole reduces LV afterload [12]. The resulting hemodynamic support is modest compared with active flow pumps or extracorporeal support [12,17].

The practical appeal of IABP includes ease of insertion, widespread availability, operator familiarity, and a generally lower vascular complication burden compared with larger-bore support devices [12]. However, contemporary guidance does not support routine IABP as a mortality-reducing strategy in unselected AMI-CS [5,6,12]. Its current role is best understood as selective rather than routine: lower-risk LV-predominant shock, bridge-to-decision, mechanical complications requiring temporization, limited vascular access options, or situations in which bleeding risk makes larger-bore support less attractive [5,6,12].

### 3.2. Microaxial Flow Pumps

Microaxial flow pumps, most commonly Impella devices, are placed across the aortic valve and pump blood from the LV into the ascending aorta [12]. This provides active LV unloading and forward systemic flow, which may reduce LV end-diastolic pressure, decrease myocardial oxygen demand, support systemic perfusion, and accelerate metabolic recovery [12,18].

The physiologic rationale is strongest in patients with STEMI-related cardiogenic shock in whom left ventricular failure is the predominant mechanism of shock [12,18,19]. The main limitation is harm from device-related complications, including bleeding, vascular injury, limb ischemia, hemolysis, suction events, acute kidney injury, renal replacement therapy, and infection [18,19,20]. Therefore, the clinical value of microaxial flow pump therapy depends on whether improved perfusion and unloading outweigh the complication burden in a carefully selected patient.

### 3.3. Venoarterial Extracorporeal Membrane Oxygenation

VA-ECMO provides both circulatory support and gas exchange by draining venous blood, oxygenating it through an extracorporeal membrane, and returning it to the arterial circulation [12,21]. This makes VA-ECMO particularly attractive for profound shock with severe hypoxemia, cardiac arrest, RV failure, or biventricular failure [12].

However, peripheral VA-ECMO increases retrograde aortic flow and may increase LV afterload [12,14,15,16]. In patients with poor LV ejection, this can worsen LV distension, pulmonary congestion, myocardial wall stress, and delayed recovery [14,15,16]. VA-ECMO also requires large-bore cannulation and systemic anticoagulation, contributing to bleeding, vascular injury, limb ischemia, stroke, infection, and prolonged mechanical ventilation [22,23,24].

## 4. Randomized Evidence

### 4.1. Intra-Aortic Balloon Pump

Older randomized data demonstrated no clear mortality benefit with routine IABP in AMI-CS, a finding reflected in contemporary guidelines and reviews [5,6,12,25]. IABP may nevertheless remain useful for selected patients requiring modest LV unloading, temporary stabilization, or when larger-bore support is unsuitable [12].

### 4.2. Microaxial Flow Pump Therapy

DanGer Shock is the pivotal contemporary randomized trial evaluating the routine use of Impella CP in patients with STEMI-related cardiogenic shock. The trial randomized selected patients with STEMI-related cardiogenic shock to routine Impella CP support plus standard care or standard care alone [19]. Routine Impella CP support reduced 180-day all-cause mortality compared with standard care alone (45.8% vs. 58.5%; HR 0.74, 95% CI 0.55–0.99) [19].

However, the trial excluded patients with mechanical complications, right ventricular failure, and comatose survivors of out-of-hospital cardiac arrest, limiting generalizability to broader AMI-CS populations and other microaxial flow pump devices [19].

Accordingly, the findings should be interpreted within the context of the specific population enrolled in DanGer Shock and should not be extrapolated to all patients with cardiogenic shock or to all microaxial flow pump devices.

The benefit in DanGer Shock came with a clear safety cost [19]. The composite safety endpoint occurred in 24.0% of patients assigned to Impella CP compared with 6.2% assigned to standard care (RR 4.74, 95% CI 2.36–9.55). Moderate or severe bleeding occurred in 21.8% versus 11.9%, limb ischemia in 5.6% versus 1.1%, and renal replacement therapy in 41.9% versus 26.7%, respectively [19]. Subsequent analyses confirmed increased bleeding and renal complications, while hemodynamic assessment demonstrated reduced vasoactive-inotropic requirements and faster lactate normalization with Impella CP [18,20,26]. These findings characterize the physiologic and safety effects of support but do not establish the mechanism responsible for the mortality benefit.

Importantly, the mortality benefit persisted despite the higher incidence of renal complications [20]. These findings support the biologic rationale for active LV unloading while emphasizing the importance of meticulous vascular access, anticoagulation, and complication surveillance [18,19,20].

A DanGer Shock hemodynamic and metabolic substudy showed that Impella CP support reduced vasoactive-inotropic requirements while maintaining hemodynamic stability and was associated with faster lactate normalization [18]. These findings provide biologic plausibility for the observed mortality benefit but should not be interpreted as establishing the mechanism responsible for improved survival [18].

Secondary analyses further refined patient selection and treatment strategy. A post hoc analysis of the DanGer Shock trial demonstrated that escalation to higher-level temporary mechanical circulatory support occurred less frequently among patients initially treated with Impella CP than with standard care. Patients requiring escalation had substantially higher mortality regardless of treatment assignment, highlighting that progression of shock despite initial therapy identifies a particularly high-risk population and underscoring the importance of selecting an appropriate initial support strategy [27].

Exploratory analyses evaluated age, baseline systolic blood pressure, symptom-to-randomization delay, and sex as potential modifiers of treatment response [28,29,30,31]. Age and baseline systolic blood pressure showed potential heterogeneity, whereas the interaction with timing was not statistically significant and no significant interaction by sex was observed [28,29,30,31]. These findings remain hypothesis-generating; no validated treatment-selection thresholds have been established.

Revascularization strategy may also influence outcomes. A prespecified DanGer Shock substudy suggested lower observed 180-day mortality with immediate multivessel PCI than culprit-only PCI, without evidence that PCI strategy modified the treatment effect of Impella CP.8 Because PCI strategy was not randomized, these findings remain hypothesis-generating and do not establish an optimal revascularization approach [8]. These findings should be interpreted in the context of the randomized CULPRIT-SHOCK evidence [9].

### 4.3. VA-ECMO and Extracorporeal Life Support

The randomized evidence for routine early VA-ECMO in AMI-CS is less favorable. ECLS-SHOCK is the largest randomized trial of early extracorporeal life support in infarct-related cardiogenic shock [24]. In that trial, early routine ECLS did not reduce 30-day all-cause mortality compared with usual medical therapy [24]. Moderate or severe bleeding and peripheral vascular complications were more frequent with ECLS [24].

One-year follow-up from ECLS-SHOCK did not demonstrate a delayed survival advantage with routine ECLS [32]. These findings do not support routine early VA-ECMO as a default strategy for broadly selected patients with AMI-CS [24,32].

The ECMO-CS trial randomized patients with severe or rapidly deteriorating cardiogenic shock to immediate VA-ECMO or an early conservative strategy that allowed downstream ECMO if needed. Immediate VA-ECMO did not improve the primary composite outcome compared with the early conservative strategy [21]. One-year follow-up also failed to show a long-term clinical advantage of immediate VA-ECMO [33].

EURO SHOCK evaluated early VA-ECMO in patients with persistent cardiogenic shock after primary PCI for acute myocardial infarction. The trial did not demonstrate a statistically significant survival advantage with early VA-ECMO, but interpretation was limited by early termination and small sample size. Bleeding and vascular complications were more frequent in the VA-ECMO arm [22].

An individual patient data meta-analysis of randomized trials found that early routine VA-ECMO did not reduce mortality in infarct-related cardiogenic shock and increased major bleeding and peripheral ischemic vascular complications [13].

A subsequent mediation analysis of ECLS-SHOCK suggested that although VA-ECMO increased bleeding and vascular complications, these adverse events did not explain the absence of a survival benefit. As a post hoc mediation analysis, these observations should be considered exploratory and hypothesis-generating rather than mechanistic proof [34].

A prespecified ECLS-SHOCK substudy compared patients managed with upfront VA-ECMO, bailout mechanical circulatory support after initial conservative therapy, and escalation to additional support following VA-ECMO implantation. Although not powered for definitive comparisons, patients requiring bailout or escalated support experienced numerically higher 30-day mortality and greater use of renal replacement therapy. These findings likely reflect greater baseline shock severity rather than harm from escalation itself, suggesting that patients requiring bailout or escalation represent an extremely high-risk population and highlighting the importance of timely patient selection and early recognition of patients who may require advanced circulatory support [35]. Because the analysis was not powered for definitive comparisons, these observations should be interpreted cautiously.

The neutral VA-ECMO findings should not be interpreted as evidence that ECMO has no role in AMI-CS. Rather, they suggest that routine early VA-ECMO for broadly selected AMI-CS patients is not supported [21,22,23,24,32,33]. VA-ECMO may still be considered in carefully selected patients with refractory circulatory collapse, severe hypoxemia, cardiac arrest with potentially reversible disease, predominant right ventricular or biventricular failure, or persistent hypoperfusion despite vasoactive therapy and less invasive support. These recommendations are based primarily on physiologic rationale, clinical experience, and current expert consensus rather than direct randomized evidence [12,21,22,24,32,33].

Post hoc analyses may help refine patient selection. The SCAI shock classification has been consistently validated as a prognostic tool in cardiogenic shock [2,3]. A prespecified ECLS-SHOCK substudy further confirmed that increasing SCAI shock severity was associated with progressively higher mortality in AMI-related cardiogenic shock36.

However, SCAI stage alone did not identify a subgroup deriving a survival benefit from routine VA-ECMO, suggesting that treatment decisions should integrate shock phenotype, hemodynamics, and end-organ dysfunction rather than shock stage alone [36]. A post hoc analysis of ECMO-CS suggested that more specific hemodynamic or metabolic markers, including low cardiac index, low venous oxygen saturation, or elevated carbon dioxide gap, may help identify patients in whom immediate VA-ECMO warrants further investigation; however, these observations remain hypothesis-generating and require prospective validation before influencing routine clinical decision-making [37].

### 4.4. LV Unloading During VA-ECMO

One major physiologic limitation of VA-ECMO is LV afterload increase [12,14,15,16,38]. When the LV cannot eject effectively, VA-ECMO may worsen LV distension and pulmonary congestion [12,14,15,16]. This has led to increasing interest in LV unloading strategies during VA-ECMO, including IABP, microaxial flow pumps, atrial septostomy, transseptal left atrial cannulation, and surgical venting [12,14,15,16].

The EARLY-UNLOAD randomized trial compared a strategy of routine early LV unloading after VA-ECMO initiation with a conventional strategy in which unloading was performed selectively as rescue therapy [14]. Early unloading did not reduce 30-day mortality, although it improved pulmonary congestion [14]. One-year follow-up similarly showed no improvement in clinical outcomes with early routine LV unloading [15].

EVOLVE-ECMO evaluated early left atrial venting compared with conventional management for LV decompression during VA-ECMO [16]. Early left atrial venting did not improve successful VA-ECMO weaning or survival to discharge, although pulmonary congestion improved [16].

Collectively, these trials demonstrated improvement in physiologic congestion markers but have not demonstrated a consistent survival benefit with routine early LV unloading in randomized studies [14,15,16]. Future studies should identify which patients have clinically harmful LV distension, which unloading approach is safest, and whether unloading guided by invasive hemodynamics, echocardiography, aortic valve opening, pulmonary edema, and lactate trajectory improves outcomes [12,14,15,16].

A recurring observation across contemporary randomized trials is that improvements in hemodynamic parameters do not necessarily translate into improved survival. Mechanical circulatory support reliably augments cardiac output, reduces ventricular filling pressures, and improves systemic perfusion; however, many patients are enrolled after extensive ischemic injury, systemic inflammation, microvascular dysfunction, and multiorgan failure have already become established. Consequently, restoration of macrocirculatory flow alone may be insufficient to reverse the complex pathophysiology of advanced cardiogenic shock. This disconnect may help explain why several devices demonstrate favorable physiologic effects despite neutral mortality outcomes in randomized trials [1,12,14,15,16,21,22,23,24,32,33].

Contemporary randomized trials have renewed interest in the timing of mechanical circulatory support in relation to the evolution of cardiogenic shock. However, available randomized studies have not established an optimal timing strategy, and whether earlier physiology-guided implementation improves survival remains an important unanswered question requiring prospective investigation [12,22].

## 5. Comparative Analysis of Mechanical Circulatory Support Devices: Lessons from Randomized Clinical Trials

### 5.1. Comparative Interpretation of Contemporary Randomized Evidence

Contemporary randomized clinical trials demonstrate that no temporary mechanical circulatory support device is universally superior for all patients with AMI-CS. Rather, successful outcomes depend on appropriate patient selection, timing of support, and multidisciplinary shock care [12,13,17,23].

### 5.2. Comparison of IABP, Microaxial Flow Pumps, and VA-ECMO

Contemporary randomized trials demonstrate a trade-off between increasing circulatory support and procedural risk. IABP provides modest hemodynamic support but has not demonstrated a routine survival benefit [25]. In contrast, Impella CP, as evaluated in the DanGer Shock trial, demonstrated improved 180-day survival in a carefully selected STEMI-related cardiogenic shock population, although with increased device-related complications [19]. Routine early VA-ECMO has not demonstrated a survival benefit across randomized trials and has consistently been associated with higher complication rates [13,24].

Earlier randomized trials of microaxial flow support, including IMPRESS and IMPELLA-STIC, were limited by small sample sizes and were not powered to demonstrate a mortality benefit [39,40,41].

Because these trials enrolled different patient populations and evaluated different treatment strategies, they should not be interpreted as direct head-to-head comparisons of MCS devices.

The principal characteristics, clinical signals, complications, and potential clinical scenarios for these devices are summarized in Table 1.

### 5.3. Lessons Learned from Randomized Trials

Collectively, contemporary randomized trials demonstrate that no temporary MCS strategy is universally beneficial in AMI-CS. Rather, outcomes depend on careful patient selection, balancing potential survival benefit against device-related complications, and integrating device selection with the underlying shock phenotype and clinical context. The contrasting findings of DanGer Shock and contemporary VA-ECMO trials emphasize that AMI-CS is a heterogeneous syndrome requiring individualized treatment strategies rather than routine device deployment [12,19,21,23].

Taken together, these randomized trials have shifted contemporary practice away from routine device deployment toward individualized implementation of temporary mechanical circulatory support based on clinical presentation, shock severity, and multidisciplinary assessment.

Clinically relevant prespecified secondary and post hoc analyses of contemporary randomized trials are summarized in Table 2.

### 5.4. Current Clinical Guidelines and Expert Consensus

Reflecting these findings, current ACC/AHA guidelines and expert consensus statements advocate an individualized approach to temporary MCS rather than routine implementation of any single device. Current guidelines and expert consensus suggest that, following comprehensive clinical and hemodynamic assessment, patients presenting predominantly with left ventricular failure may be considered for microaxial flow pump support, whereas VA-ECMO may be considered in selected patients with refractory circulatory collapse, severe hypoxemia, or biventricular failure. IABP continues to serve a selective role in patients requiring modest circulatory support, temporary stabilization, mechanical complications of myocardial infarction, or adjunctive ventricular unloading. Contemporary practice increasingly emphasizes multidisciplinary shock teams, invasive hemodynamic assessment, and serial reassessment to guide escalation or de-escalation of support according to the patient’s evolving clinical status rather than adherence to a fixed device algorithm [3,5,6,12,23].

The major randomized trials, including differences in study populations, timing of support and revascularization, efficacy outcomes, safety findings, and crossover or bailout strategies, are summarized in Table 3.

## 6. Future Directions

Future research should focus on integrated strategies rather than isolated devices. Important questions include early microaxial flow pump support versus selective escalation, VA-ECMO with protocolized LV unloading versus VA-ECMO alone, ECMO-first versus Impella-first pathways in specific phenotypes, and staged revascularization timing after culprit-only PCI [12,13,14,15,16,23].

Patient selection should be improved through prospective shock phenotyping. Trials should define LV-predominant, RV-predominant, biventricular, hypoxemic, post-arrest, and hemometabolic shock subgroups before randomization [1,3,12]. Invasive hemodynamics may help identify patients more likely to benefit from a specific support strategy [12,18]. Low cardiac index, low venous oxygen saturation, high filling pressures, pulmonary artery pulsatility index, pulmonary capillary wedge pressure, lactate trajectory, carbon dioxide gap, and vasoactive-inotropic score may be more informative than blood pressure alone [12,18].

Timing should also be treated as a central trial variable. MCS placed too late may not reverse established multiorgan failure, while MCS placed too early may expose patients who would stabilize after PCI to unnecessary harm [12,21,22,24,32,33]. The optimal timing is likely device- and phenotype-specific.

Complication-reduction protocols should be standardized in future trials. These should include ultrasound-guided access, preclosure strategies, distal perfusion catheters when appropriate, anticoagulation targets, hemolysis monitoring, limb perfusion surveillance, renal protection strategies, daily assessment of device necessity, and structured weaning protocols [12,19,20,22,24].

Finally, future endpoints should include more than short-term survival. Clinically meaningful outcomes include survival with acceptable neurologic function, renal recovery, freedom from dialysis, ventilator-free days, lactate clearance, vasoactive de-escalation, cardiac recovery, rehospitalization, quality of life, and functional status.

## 7. Limitations

This review has several limitations. First, although contemporary randomized trials provide the highest level of evidence, important differences in patient populations, shock severity, cardiac arrest status, revascularization strategies, timing of mechanical circulatory support, and definitions of adverse events limit direct comparison across studies. Second, many clinically relevant observations derive from prespecified secondary or post hoc analyses and should therefore be interpreted as hypothesis-generating. Third, because this is a narrative review, the literature selection was based on clinical relevance rather than systematic evidence synthesis. Finally, rapid evolution of temporary mechanical circulatory support technologies and ongoing randomized trials may further refine current treatment strategies.

## 8. Conclusions

Recent randomized evidence has refined the role of temporary mechanical circulatory support in AMI-CS. Routine IABP is not supported as a universal mortality-reducing strategy, although it remains useful in selected lower-risk or bridge-to-decision scenarios. Routine early VA-ECMO does not improve survival in broad infarct-related cardiogenic shock populations and increases bleeding and vascular complications. Impella CP, as evaluated in the DanGer Shock trial, currently provides the strongest randomized survival signal, but this evidence applies to carefully selected patients with LV-predominant STEMI-CS treated in experienced systems.

The future of MCS in AMI-CS is not simply more device use. It is better matching of device physiology to shock phenotype, earlier recognition of reversible shock before irreversible organ injury, safer implantation, standardized multidisciplinary shock-team protocols, and structured weaning guided by hemodynamics and end-organ recovery.

## Figures and Tables

**Table 1:** Comparative Summary of Mechanical Circulatory Support Devices in AMI-CS.

Device	Main Mechanism	Recent Randomized Evidence	Main Clinical Signal	Main Complications	Potential Clinical Scenario
IABP	Diastolic augmentation and systolic afterload reduction	Contemporary guideline interpretation and MCS reviews [5,6,12]	No clear routine mortality benefit in broad AMI-CS [5,6,12]	Generally lower vascular and bleeding burden than larger-bore devices [12]	Lower-risk LV-predominant shock; bridge-to-decision; limited access options [12]
Microaxial flow pump	Direct LV unloading and forward flow from LV to aorta	DanGer Shock and secondary analyses [18,19,20,28,29]	Reduced 180-day mortality in selected STEMI-CS; faster metabolic recovery [18,19]	Bleeding, limb ischemia, hemolysis, AKI, renal replacement therapy, sepsis [19,20]	Potential clinical scenario based on physiologic rationale and the DanGer Shock trial population [18,19,20,28,29]
VA-ECMO/ECLS	Full cardiopulmonary support with oxygenation and systemic perfusion	ECLS-SHOCK, ECMO-CS, EURO SHOCK, IPD meta-analysis [21,22,23,24,32,33]	No routine mortality benefit in broad AMI-CS trial populations [21,22,23,24,32,33]	Major bleeding, vascular injury, limb ischemia, stroke, infection, LV afterload increase [22,23,24]	Profound refractory shock, severe hypoxemia, RV/biventricular failure, potentially reversible cardiac arrest [12,21,22,24,32,33]

**Table 2:** Clinically Relevant Prespecified Secondary and Post Hoc Analyses of Contemporary Randomized Trials of Temporary Mechanical Circulatory Support in AMI-Related Cardiogenic Shock.

Analysis	Parent Trial	Analysis Type	Main Finding	Clinical Implication	Interpretation
Hemodynamic and metabolic effects	DanGer Shock	Substudy	Impella CP reduced vasoactive-inotropic requirements while maintaining hemodynamic stability and was associated with faster lactate normalization.	Provides physiologic support for improved perfusion and LV unloading with Impella CP.	Mechanistic findings; do not establish the mechanism responsible for the mortality benefit. Ref. [18]
Renal outcomes	DanGer Shock	Secondary analysis	AKI and renal replacement therapy were more frequent with Impella CP, although the mortality benefit observed in the parent trial persisted.	Highlights renal complications as an important component of the benefit–risk balance and the need for renal monitoring.	Secondary analysis; should not be used independently for treatment selection. Ref. [20]
Bleeding outcomes	DanGer Shock	Substudy	Major bleeding increased with increasing complexity of MCS; half of bleeding events occurred after device removal.	Emphasizes vascular-access management and continued surveillance after device removal.	Supports complication-mitigation strategies rather than defining candidacy for Impella CP. Ref. [26]
Escalation of mechanical circulatory support	DanGer Shock	Post hoc analysis	Escalation to higher-level temporary MCS occurred less frequently after initial Impella CP than with standard care; patients requiring escalation had substantially higher mortality.	Failure of the initial support strategy identifies an especially high-risk population.	Hypothesis-generating; escalation itself should not be inferred to cause the higher mortality. Ref. [27]
Age and treatment response	DanGer Shock	Exploratory post hoc analysis	Possible heterogeneity in the effect of Impella CP according to age was observed.	Age may contribute to patient selection, but no clinically validated cutoff has been established.	Hypothesis-generating; should not be used as an age-based treatment threshold. Ref. [28]
Baseline systolic blood pressure	DanGer Shock	Exploratory analysis	Baseline SBP was evaluated as a potential modifier of the treatment effect of Impella CP.	Hemodynamic phenotype may be more informative than a single diagnosis when considering MCS.	Exploratory; requires prospective validation before use in routine treatment selection. Ref. [29]
Delay from symptom onset to treatment	DanGer Shock	Exploratory analysis	Increasing delay from symptom onset to randomization suggested attenuation of treatment effect, but the interaction was not statistically significant.	Timing of support remains an important research question.	Does not establish an optimal implantation window or support a specific timing strategy. Ref. [30]
Sex-specific outcomes	DanGer Shock	Prespecified secondary analysis	Women were older and presented later than men and had higher mortality; no significant interaction between sex and treatment assignment was demonstrated.	Presentation characteristics may contribute to sex-related outcome differences.	Does not support sex-specific selection for Impella CP; findings remain exploratory. Ref. [31]
Multivessel coronary disease and revascularization	DanGer Shock	Secondary analysis	Among patients with multivessel coronary artery disease, immediate multivessel PCI was associated with lower observed 180-day mortality than culprit-only PCI; however, PCI strategy did not modify the treatment effect of Impella CP.	Revascularization strategy may influence outcomes and should be considered when comparing MCS trials.	Nonrandomized comparison of PCI strategy; hypothesis-generating and not evidence for a preferred revascularization approach. Ref. [8]
Upfront, bailout, and escalated MCS	ECLS-SHOCK	Prespecified substudy	Patients requiring bailout MCS after conservative therapy or escalation after VA-ECMO had numerically higher mortality and greater use of renal replacement therapy.	Need for bailout or escalation identifies a population with particularly severe or progressive shock.	Not powered for definitive between-group comparisons; likely reflects baseline/sequential shock severity rather than harm from escalation itself. Ref. [35]
SCAI shock severity	ECLS-SHOCK	Prespecified substudy	Increasing SCAI shock severity was associated with progressively higher mortality, but SCAI stage did not identify a subgroup with a survival benefit from routine VA-ECMO.	SCAI stage is useful for prognosis but should not be used alone to determine VA-ECMO candidacy.	Supports integration of shock stage with phenotype, hemodynamics, and end-organ dysfunction. Ref. [36]
Mediation of the neutral VA-ECMO effect	ECLS-SHOCK	Post hoc mediation analysis	Although VA-ECMO increased bleeding and vascular complications, these adverse events did not explain the absence of a mortality benefit.	Neutral mortality with routine VA-ECMO cannot be attributed solely to device-related complications.	Exploratory mediation analysis; does not establish a causal mechanism for the neutral trial result. Ref. [34]
Hemodynamic and metabolic predictors of response	ECMO-CS	Post hoc analysis	Low cardiac index, low venous oxygen saturation, and an elevated venoarterial CO_2_ gap were suggested as potential markers associated with differential response to immediate VA-ECMO.	More granular hemodynamic and metabolic phenotyping may help refine future VA-ECMO selection.	Hypothesis-generating; requires prospective validation before influencing routine clinical decision-making. Ref. [37]

**Table 3:** Randomized Clinical Trials of Temporary Mechanical Circulatory Support in AMI-Related Cardiogenic Shock.

Trial	Year	N	Population/Shock Definition	Intervention	Comparator	Timing of MCS/Revascularization	Primary Endpoint	Main Efficacy (Effect Estimate)	Major Safety Outcomes	Crossover/Bailout	Key Limitations
IABP-SHOCK II	2012	600	AMI-CS undergoing early revascularization	Routine IABP	Standard care	After revascularization	30-day mortality	No mortality benefit (RR 0.96, 95% CI 0.79–1.17)	No significant increase in major complications	Limited crossover	Pre-contemporary era; before newer MCS devices
IMPRESS	2017	48	Severe AMI-CS requiring MCS	Impella CP	IABP	Early after shock	30-day mortality	No mortality benefit HR 0.96 (95% CI 0.42–2.18; *p* = 0.92)	Bleeding 33% vs. 8%; vascular complications 4% vs. 0%; hemolysis requiring device removal 8% vs. 0%	Rescue therapy permitted	Underpowered; very small sample
IMPELLA-STIC	2021	15 randomized	STEMI-CS	Impella LP5.0 + IABP	IABP	Early	Feasibility/clinical outcomes	ΔCPI: 0.08 ± 0.08 vs. −0.02 ± 0.25 W/m^2^ (*p* = 0.40)—no significant difference	More vascular complications	-	Pilot study
DanGer Shock	2024	355	Selected STEMI-CS without mechanical complications, RV failure, or comatose OHCA	Routine Impella CP + standard care	Standard care	Before/after PCI or ≤12 h post-cath; Impella CP initiated after randomization.	180-day all-cause mortality	HR 0.74 (95% CI 0.55–0.99); mortality 45.8% vs. 58.5%	Composite safety 24.0% vs. 6.2%; bleeding 21.8% vs. 11.9%; limb ischemia 5.6% vs. 1.1%; RRT 41.9% vs. 26.7%	Escalation allowed	Highly selected population; limited generalizability
ECMO-CS	2023	117	Severe or rapidly deteriorating cardiogenic shock	Immediate VA-ECMO	Early conservative strategy	Immediate	Composite endpoint	HR 0.72 (95% CI 0.46–1.12), *p* = 0.21 for the primary endpoint (63.8% vs. 71.2%). No significant benefit	Bleeding 31.0% vs. 20.3%; limb ischemia 13.8% vs. 5.1%; stroke 5.2% vs. 0%	Bailout ECMO permitted	Mixed shock etiologies
EURO SHOCK	2023	35	Persistent AMI-CS after primary PCI	Early VA-ECMO	Standard care	After PCI	30-day mortality	HR 0.56 (95% CI 0.21–1.45); *p* = 0.22; 43.8% vs. 61.1% mortality. No significant mortality benefit	Bleeding 35.7% vs. 5.6%; vascular complications 21.4% vs. 0%	-	Early termination; small sample
ECLS-SHOCK	2023	420	Infarct-related cardiogenic shock	Early routine ECLS	Usual care	Early after randomization	30-day mortality	RR 0.98 (95% CI 0.80–1.19); no mortality benefit	Bleeding 23.4% vs. 9.6%; peripheral vascular complications 11.0% vs. 3.8%; stroke 3.8% vs. 2.9%	Rescue MCS permitted	Routine strategy evaluated in heterogeneous population
EARLY-UNLOAD	2023	116	VA-ECMO patients	Routine early LV unloading	Conventional strategy (rescue unloading allowed)	Within hours of ECMO	30-day mortality	HR 1.02 (95% CI 0.59–1.74; *p* = 0.942); 30-day mortality 46.6% vs. 44.8%. No mortality benefit	BARC 3/5 bleeding 10.3% vs. 13.8%; limb ischemia 5.2% vs. 6.9%; stroke 10.3% vs. 8.8%	Rescue unloading allowed	Compared unloading strategy, not ECMO itself
EVOLVE-ECMO	2023	60	VA-ECMO requiring LV decompression	Early left atrial venting	Conventional management	Early	Successful ECMO weaning	70.0% vs. 76.7%; RR 0.91 (95% CI 0.67–1.24), *p* = 0.386. No improvement in survival or weaning	Puncture-site bleeding 20.0% vs. 16.7%; limb ischemia 13.3% vs. 13.3%; CRRT 63.3% vs. 60.0%	-	Single unloading strategy; modest sample

Abbreviations: AKI, acute kidney injury; AMI-CS, acute myocardial infarction-related cardiogenic shock; ECLS, extracorporeal life support; IABP, intra-aortic balloon pump; LV, left ventricle; MCS, mechanical circulatory support; OHCA, out-of-hospital cardiac arrest; PCI, percutaneous coronary intervention; RR, relative risk; RRT, renal replacement therapy; RV, right ventricle; STEMI, ST-segment elevation myocardial infarction; VA-ECMO, venoarterial extracorporeal membrane oxygenation. **Note:** Definitions and ascertainment of adverse events differed across trials; therefore, direct quantitative comparisons of safety outcomes between studies should be interpreted with caution.

## Data Availability

No new data were created or analyzed in this study. Data sharing is not applicable to this article.
